# “Picture this from there”: spatial perspective-taking in developmental visuospatial disorder and developmental coordination disorder

**DOI:** 10.3389/fpsyg.2024.1349851

**Published:** 2024-04-19

**Authors:** Camilla Orefice, Ramona Cardillo, Isabella Lonciari, Leonardo Zoccante, Irene C. Mammarella

**Affiliations:** ^1^Department of Developmental and Social Psychology, School of Psychology, University of Padua, Padua, Italy; ^2^Department of Women’s and Children’s Health, School of Medicine and Surgery, University of Padua, Padua, Italy; ^3^Division of Child Neurology and Psychiatry, University Pediatric Hospital “IRCCS Burlo Garofolo”, Trieste, Italy; ^4^Child and Adolescent Neuropsychiatry Unit, Maternal-Child Integrated Care Department, Integrated University Hospital of Verona, Verona, Italy

**Keywords:** visuospatial perspective-taking, visuospatial processing, fine-motor skills, developmental visuospatial disorder, developmental coordination disorder

## Abstract

**Introduction:**

Either Developmental Visuospatial Disorder (DVSD) and Developmental Coordination Disorder (DCD) present with difficulties in visuospatial processing, even though entailing different degrees of impairment. Among the visuospatial domain, spatial perspective taking is essential to interact with the environment and is significantly involved in many daily activities (e.g., environment navigation and spatial orienting). Notwithstanding, no previous studies have investigated this spatial domain in children with DVSD and limited evidence is available regarding DCD. Consistent with a transdiagnostic approach, the first goal of the present study was to compare spatial perspective taking abilities of these groups, also including a control group of not diagnosed peers (ND). Secondly, the role of different fine-motor and visuo-spatial predictors on the spatial perspective taking performance was considered.

**Method:**

A total of 85 participants (DVSD = 26; DCD = 26; ND = 33), aged between 8 and 16 years old, were included in the study. Tasks assessing spatial perspective taking, fine-motor, visual imagery, and mental rotation skills, as well as visuo-spatial working memory were administered.

**Results and Discussion:**

Overall, our results confirmed weaknesses in spatial perspective taking in both clinical groups, with the DVSD obtaining the lowest scores. Similarities and differences in the predictors accounting for the performance in the spatial perspective taking task emerged, suggesting the possible employment of different fine-motor or visuospatial strategies by group. Findings are discussed considering the potential impact they may have both in research and clinical practice.

## 1 Introduction

### 1.1 Visuospatial deficits in DVSD and DCD

Among Neurodevelopmental Disorders, more than one condition presents with visuo-spatial deficits or with atypical visuospatial processing (World Health Organization, 1993; Caron et al., 2006; Fisher et al., 2022; Zoia et al., 2022).

Developmental Visuospatial (Non-Verbal) Disorder (DVSD) is a condition whose main feature is a massive and persistent impairment in several dimensions of visuospatial processing, for instance visuospatial memory, three-dimensional thinking, and spatial estimation, determining impairments in adaptive and everyday functioning (Mammarella and Cornoldi, 2020; Fisher et al., 2022; Mammarella et al., 2023). In addition, anecdotical evidence reports this population as clumsy (Broitman et al., 2020). Accordingly, research has suggested that children with DVSD may present with motor difficulties, mainly in the manual dexterity domain (Nichelli and Venneri, 1995; Durand, 2005; Semrud-Clikeman et al., 2010), although more research is needed to better define this domain (Mammarella, 2020; Fisher et al., 2022). Associated difficulties in mathematics and in school disciplines relying on visuospatial processing or visuo-motor integration are often reported (Mammarella et al., 2010, 2013, 2023), as well as social, emotional or behavioral difficulties (Fisher et al., 2022). To the best of our knowledge, only one study has endeavored to estimate the prevalence of the DVSD cognitive profile. This study unveiled a range of 3 to 4% in the general population (Margolis et al., 2020). Notwithstanding DVSD currently being unrecognized by the diagnostic nomenclatures (i.e., DSM, ICD), leading to misdiagnoses and lack of intervention (Mammarella and Cornoldi, 2014), research is progressing aiming to better define the disorder’s features and foster its inclusion in subsequent editions of the diagnostic manuals (Fisher et al., 2022). Among the first steps toward this aim, two Consensus Conferences were held at the Columbia University in 2017 and 2018 (Broitman et al., 2020). Following the works of the committee, a set of shared criteria has been defined and in 2022 a proposal to include DVSD in future versions of the manual has been submitted to the DSM Steering committee (The NVLD Project, 2022).

Along with DVSD, Developmental Coordination Disorder (DCD) may present with difficulties in visuospatial processing, although huge heterogeneity is reported (Cardillo et al., 2024). More in depth, some studies concluded for the presence of visuospatial impairments in this population (e.g., Van Waelvelde et al., 2004; Tsai et al., 2008; Gras et al., 2023), while others did not find any impairment (i.e., Bonifacci, 2004). Such heterogeneity in performances, albeit being reconducible to intra-group variability, well documented in neurodevelopmental disorders (e.g., Van Waelvelde et al., 2004; Tsai et al., 2008), may be attributed to methodological differences in the studies. In fact, from the analysis of the extant literature often emerged small sample sizes (e.g., Ameratunga et al., 2004; King et al., 2011), heterogeneous age ranges (e.g., Ameratunga et al., 2004; Crawford and Dewey, 2008; King et al., 2011; Tsai et al., 2012; Pisella et al., 2020), as well as variability in the method of identification of children with or without DCD (i.e., use of different cut-off scores or lack of confirmation of the diagnosis using shared criteria) (e.g., Van Waelvelde et al., 2004; Williams et al., 2006; Crawford and Dewey, 2008; Tsai et al., 2008, 2012; Wang et al., 2017). Nonetheless, Gras et al. (2023) pointed out that the presence of visuospatial difficulties in the DCD profile seems to constitute a marker for a more severe condition than the presence of motor difficulties alone. In any case, DCD is primarily defined by the presence of an “*impairment in the execution of coordinated motor skills*,” as suggested by the ICD-11 nomenclature (World Health Organization, 2018). In this regard, it is important to notice a change in the nomenclature of the disorder from the ICD-10 (World Health Organization, 1993) to the ICD-11 (World Health Organization, 2018). In this latter version it is referred to Developmental Motor Coordination Disorder. However, to avoid any ambiguity on the actual diagnosis, Blank et al. (2019) suggested to use the term DCD; therefore, in the present work we adhere to this principle. Children receiving a DCD diagnosis encounter severe difficulties in the acquisition and in the execution of motor skills, in which they perform far below the expected level according to age and learning opportunities. Moreover, these difficulties significantly interfere with their everyday functioning, leading to challenges in several domains (i.e., school learning and play) (American Psychiatric Association, 2013). Recent research has suggested that the typical symptoms of DCD are not limited to the motor aspects (Blank et al., 2019), entailing possible consequences also in the socio-emotional (Zwicker et al., 2013; Sumner et al., 2016; Blank et al., 2019) as well as in the cognitive (Green et al., 2008; Schoemaker et al., 2013; Sumner et al., 2016) domains.

As well documented in neurodevelopmental disorders, and as emerges from the previous description, although DVSD and DCD constitute discrete disorders (Blank et al., 2019; Fisher et al., 2022) they may present with overlapping symptoms (Astle et al., 2022), even though entailing different degrees of impairment.

### 1.2 Visuospatial processing

In light of the predominant role covered by visuospatial processing skills in various aspects of everyday life (Hegarty and Waller, 2005; Jansen et al., 2010), as well as in academic learning (Newcombe, 2017; Hodgkiss et al., 2018), it appears of fundamental relevance deepening the knowledge on this domain (Mix et al., 2018). To achieve this, it is essential to examine potential differences and overlapping that may occur in individuals with neurodevelopmental disorders (Volden, 2013; Astle et al., 2022), specifically in those marked by visuospatial challenges. Despite many attempts to provide a definition of visuospatial skills (i.e., Ungerleider and Mishkin, 1982; Linn and Petersen, 1985; Uttal et al., 2013; Rimfeld et al., 2017), to date there is no evidence for a shared conception nor for a definition of these abilities, as well as for an account of the underlying processes (Malanchini et al., 2020; Munns et al., 2022). However, several models have been developed aiming at disentangling this complexity. For instance, one approach distinguishes between small-scale as compared to large-scale (or environmental-scale) spatial abilities (Munns et al., 2022). The former comprises all the abilities that allow manipulation of objects smaller than the body, applied without modifications of the vantage point; on the other hand, the latter relies on the manipulation of information tackling spatial features or implying spatial imagery, requiring changes in perspective as well. In this context, albeit in the presence of huge individual differences, Hegarty et al.’s seminal work suggests a partial dissociation between small- and large-scale spatial abilities. This result aligns with prior observations made in both behavioral sciences and neuroscience (Hegarty et al., 2006). Another model, proposed by Uttal et al. (2013) relies on two main distinctions (i.e., intrinsic vs. extrinsic, dynamic vs. static). Intrinsic skills refer to an object’s features and entail defining a particular object based on the arrangement and relations of its parts, while extrinsic skills involve the relationships between-objects, whether in relation to each other or within an overarching framework. On the other hand, static skills are based on a fixed mental representation, while dynamic skills involve objects’ movement or transformation for the task to be performed (Newcombe and Shipley, 2015).

#### 1.2.1 Visuospatial perspective-taking

Analyzing the common points between the two models that were described above, visuospatial perspective-taking (VSPT) emerges as an example of the large-scale/environmental spatial abilities (Münzer et al., 2018; Munns et al., 2022) and, at the same time, could be considered among the extrinsic-dynamic spatial skills according to Uttal et al.’s (2013) model (Mix et al., 2018). However, it is worth noting that other authors are prone to consider VSPT among the small-scale abilities (Fields and Shelton, 2006; Meneghetti et al., 2014, 2021). The matter is further complicated by Hegarty et al.’s (2006) conclusion, in which the authors claim that perspective taking skills may serve as mediators of the relation between small- and large-scale spatial abilities. The importance of VSPT in everyday functioning, for human interaction and socialization has been acknowledged in research dating back to Piaget (Piaget and Inhelder, 1956), and continues to be a significant focus (Samuel et al., 2023). VSPT has been found to be linked to everyday functioning (Frick and Baumeler, 2017) and to be related, for instance, to domains like navigation and wayfinding (Kozhevnikov et al., 2006), but also to social cognition (Hamilton et al., 2014; Tanaś and Myslinska Szarek, 2021). Despite its significance across a range of abilities, ranging from socialization to spatial navigation, and despite extensive research over the decades, a comprehensive theory of VSPT has yet to be developed and findings are often heterogeneous (Samuel et al., 2023). Samuel et al. (2023) attempted to shed light on this controversial construct and concluded that this heterogeneity may arise from the adoption of different strategies during VSPT tasks, as well as from actual individual differences in VSPT skills. Finally, the authors pointed out that some context-specific factors (e.g., culture, bilingualism, cognitive skills such as executive functions) could influence performance. Moving on to consider possible differences in VSPT tasks, research has suggested a possible classification of visuospatial perspective taking into two different levels (i.e., VSPT level 1 and VSPT level 2) (Flavell, 1977). In VSPT level 1 (L-1, also referred to as perspective-tracking) it is required to monitor another one’s point of view, without changing the vantage point. On the other hand, VSPT level 2 (L-2) requires to assume a different viewpoint (Surtees et al., 2013; Tanaś and Myslinska Szarek, 2021). Accordingly, L-2 VSPT could be defined as the ability to mentally imagine a scene from an external viewpoint, taking into account the relative position of all the elements that are involved in Zacks et al., (2003) and Kessler and Thomson (2010), as well as the multifaceted different perspectives that may concur to define the scene’s features (Pearson et al., 2013). Perspective-tracking is said to emerge at the age of 2, while level-2 VSPT seems a diverse cognitive process, more difficult to perform, and becomes apparent around 4–5 years of age (Newcombe, 1989; Moll and Tomasello, 2006; Gunia et al., 2021). Based on recent research, level-2 VSPT is an embodied process (Kessler and Rutherford, 2010; Kessler and Thomson, 2010), implying the engagement of two types of embodiment, spatial and motor (Amorim et al., 2006; Gunia et al., 2021).

The short Object Perspective-Taking Test (sOPT, adapted from Kozhevnikov and Hegarty, 2001; Hegarty and Waller, 2004) is a task commonly used for the assessment of VSPT (L-2 VPT). Previous studies have shed light on the role of several visuospatial and motor skills in determining the performance in the sOPT Test. Some of them pointed out the association between the performance in the sOPT Test and either visual and spatial imagery abilities (Kessler and Rutherford, 2010; Meneghetti et al., 2012; Cardillo et al., 2020). On the other hand, some other studies highlighted the strict relationship between visuospatial perspective-taking and mental rotation abilities (Kozhevnikov and Hegarty, 2001; Hegarty and Waller, 2004; Esenkaya et al., 2017). In addition, visuospatial working memory has been found to be associated to the sOPT performance (Meneghetti et al., 2012; Cardillo et al., 2020). Associations between visuospatial perspective-taking and the motor domain have also been documented, involving both fine- (Cardillo et al., 2020) and gross-motor (Hötting et al., 2021) abilities.

Given the link between VSPT and the aforementioned domains and its crucial role in children’s everyday functioning, studying perspective-taking abilities in atypical populations could provide important information on their cognitive functioning, as well as serve as a hint in designing treatment and habilitative plans. Strikingly, no previous studies have investigated this spatial domain in children with DVSD and very limited evidence is available regarding DCD. Gauthier et al. (2018) administered, to a group of children diagnosed with DCD and to peers without any diagnosis, a dynamic imitative task, in which visuospatial perspective-taking abilities were involved, along with motor and imitative skills. In particular, they administered a 3D adaptation of the Tightrope Walker Paradigm (Thirioux et al., 2009), whose aim is the assessment of the inhibition of the egocentric perspective and of the ability to assume others’ perspective. Overall, their results suggest an impairment in VSPT for the DCD group, which scored lower than the typically developing peers; however, the authors highlighted visuospatial difficulties in the clinical group, suggesting their possible effect on the performance on the VSPT task, in combination with motor impairments.

### 1.3 The present study

The state of the art leads to the need to analyze visuospatial perspective-taking skills in DVSD and DCD. Stated the partial overlapping symptoms between the two profiles, it appears relevant to consider the role that different visuospatial skills, as well as motor abilities, may cover in supporting VSPT skills for each group. Thus, the present study aimed to analyze VSPT abilities in children diagnosed with DVSD or DCD, comparing them with a group of not-diagnosed peers (ND). To do so, participants were administered with paper-and-pencil tasks aimed at assessing possible predictors, selected among the visuospatial and the motor domains that had been proven to be linked with VSPT.

Considering the exploratory nature of this study, our primary goal was to investigate possible differences and similarities between the three groups (i.e., DVSD, DCD, and ND) in visuospatial perspective-taking, as well as in visuospatial processing (i.e., visual imagery, mental rotation, and visuospatial working memory) and fine-motor measures. Additionally, we aimed to analyze the processes underlying and contributing to VSPT skills within each of these groups.

In line with previous data on visuospatial abilities in the DVSD profile, we expect this group to obtain the lowest scores, as compared to the ND group, in all the measures assessing visuospatial processing, including VSPT (Fisher et al., 2022; Mammarella et al., 2023). On the other hand, we expect to find more heterogeneous performances for the DCD group in tasks assessing visuospatial processing (Gras et al., 2023). As regards the comparison between the two clinical groups (i.e., DVSD and DCD), although considering the lack of studies that have directly compared these populations, based on recent findings (Cardillo et al., 2024), we expect to find a more severe impairment in the visuo-spatial domain for the DVSD group as compared to the DCD peers. Finally, based on previous research, we expect to find comparable fine-motor skills in the two clinical groups, performing lower than the ND peers (American Psychiatric Association, 2013; Fisher et al., 2022; Cardillo et al., 2024).

Given the lack of studies that directly compared DVSD and DCD in the context of VSPT skills, we were unable to formulate specific hypotheses regarding the predictors that may account for the performance of the two clinical groups in the sOPT test. However, stated the role that visuospatial and motor skills cover in sustaining VSPT performance, we might expect to find significant effects of visual imagery abilities (Kessler and Rutherford, 2010), mental rotation (Kozhevnikov and Hegarty, 2001; Hegarty and Waller, 2004; Esenkaya et al., 2017), visuospatial working memory (Meneghetti et al., 2012) and fine-motor abilities (Cardillo et al., 2020).

## 2 Method

### 2.1 Participants

A total of 85 participants (57M, 28F), aged between 8 and 16 years old (hereafter, “children”), took part in the study. The sample comprised children diagnosed with DVSD (*N* = 26) or DCD (*N* = 26), as well as children without any diagnosis (not-diagnosed, ND, *N* = 33).

Children diagnosed with either DVSD or DCD were recruited through the collaboration of local specialized clinical services. A Full Scale Intelligence Quotient (FSIQ) above 80, as measured with the Wechsler Intelligence Scale for Children – Fourth Edition (WISC-IV, Wechsler, 2003), was required. Moreover, the Vocabulary subtest from the WISC-IV (Wechsler, 2003) was administered to all participants, aiming to confirm their verbal skills. All participants were native Italian speakers and had normal or corrected-to-normal vision and hearing; none of them had neurological, genetic, or psychopathological conditions.

Participants in the two clinical groups (i.e., DVSD and DCD) and in the ND group were matched for demographics characteristic (i.e., age and gender), as well as for verbal skills. The three groups were not statistically different regarding chronological age [*F*(2,82) = 2.304, *p* = 0.106, *η^2^_p_* = 0.053], gender distribution [*χ^2^* (2, *N* = 85) = 3.589, *p* = 0.166, *CramerV* = 0.206], nor vocabulary abilities [*F*(2,80) = 0.544, *p* = 0.583, *η^2^_p_* = 0.013].

Participants with DVSD had received independent clinical diagnoses by private psychologists or child psychiatrists at clinical specialized centers, according to the recommendations from the literature (Mammarella and Cornoldi, 2020). Moreover, agreement between two of the authors (I.C.M. and R.C.) on the inclusion of each participant was considered necessary. Finally, the Rey-Osterrieth Complex Figure Test (ROCFT; Rey, 1941, 1968) was administered, in order to confirm the DVSD clinical profiles by highlighting the presence of clinically significant weaknesses in visuospatial processing.

Children with DCD had a previous independent clinical diagnosis, according to DSM 5 (American Psychiatric Association, 2013) or ICD-10 (World Health Organization, 1993) criteria, made by private psychologists or child psychiatrists at clinical specialized centers. Their diagnoses were confirmed administering the Movement Assessment Battery for Children – Second Edition (MABC-2, Henderson et al., 2007), which allowed to highlight the presence of marked deficits in all the assessed domains (i.e., Manual Dexterity, Aiming and Catching, Balance), as well as total scaled scores within the clinical range.

Children with a DVSD or a DCD diagnosis were not included in the sample if their scores at the screening measures were suggestive of the co-occurring presence of massive visuospatial and motor impairments. For this reason, three children with a DCD diagnosis and two with a DVSD diagnosis were not enrolled in the study after the administration of the screening measures.

Not-diagnosed children were recruited among local schools and/or word-of-mouth. The presence of DVSD and DCD was excluded by administering these participants both the ROCFT (Rey, 1941, 1968) and the MABC-2 (Henderson et al., 2007).

In Table 1 are summarized the sample’s characteristics, as well as the results of the screening measures. In this regard, it is important to note that the screening measures have not been considered in the subsequent analysis, in order to avoid circularity.

**Table 1:** Characteristics of the groups with Developmental Visuospatial Disorder (DVSD), Developmental Coordination Disorder (DCD), and without any diagnosis (not-diagnosed, ND).

Measures	DVSD (*N* = 26) M (SD)	DCD (*N* = 26) M (SD)	ND (*N* = 33) M (SD)	Group significance^a^
Chronological age (months)	144.73 (31.05)	138.19 (28.04)	153.33 (22.89)	N.S.
Gender (M:F)	17:9	21:5	19:14	N.S.
Vocabulary^b^	11.50 (2.53)	11.25 (2.72)	10.88 (2.01)	N.S.
ROCFT: copy accuracy^c^	−2.70 (2.15)	−1.30 (1.68)	−0.50 (1.72)	DVSD < DCD, ND
ROCFT: recall accuracy^c^	−2.31 (1.87)	−1.45 (1.45)	−0.25 (1.44)	DVSD < DCD, ND
MABC-2: manual dexterity^d^	3.15 (2.88)	4.08 (2.53)	9.36 (2.75)	DVSD, DCD < ND
MABC-2: aiming and catching^d^	7.65 (3.12)	5.65 (3.52)	10.24 (2.56)	DCD < DVSD < ND
MABC-2: balance^d^	8.35 (3.43)	4.50 (2.52)	11.33 (2.09)	DCD < DVSD < ND

^a^Only significant comparisons are reported.

^b^Scaled scores on the Vocabulary subtest from the WISC-IV (Wechsler, 2003).

^c^ROCFT: Rey-Osterrieth Complex Figure Test (Rey, 1941, 1968), performances expressed as z scores.

^d^MABC-2 (Henderson et al., 2007), performances expressed as scaled scores.

The study was approved by the Institutional Review Board at the University of Padua, Italy. Children’s participation in the study was conditioned upon their parents or legal caretakers signing an informed consent form; children were also explicitly asked to express their agreement to the participation.

### 2.2 Materials

#### 2.2.1 Visuospatial perspective-taking

**Figure 1:**
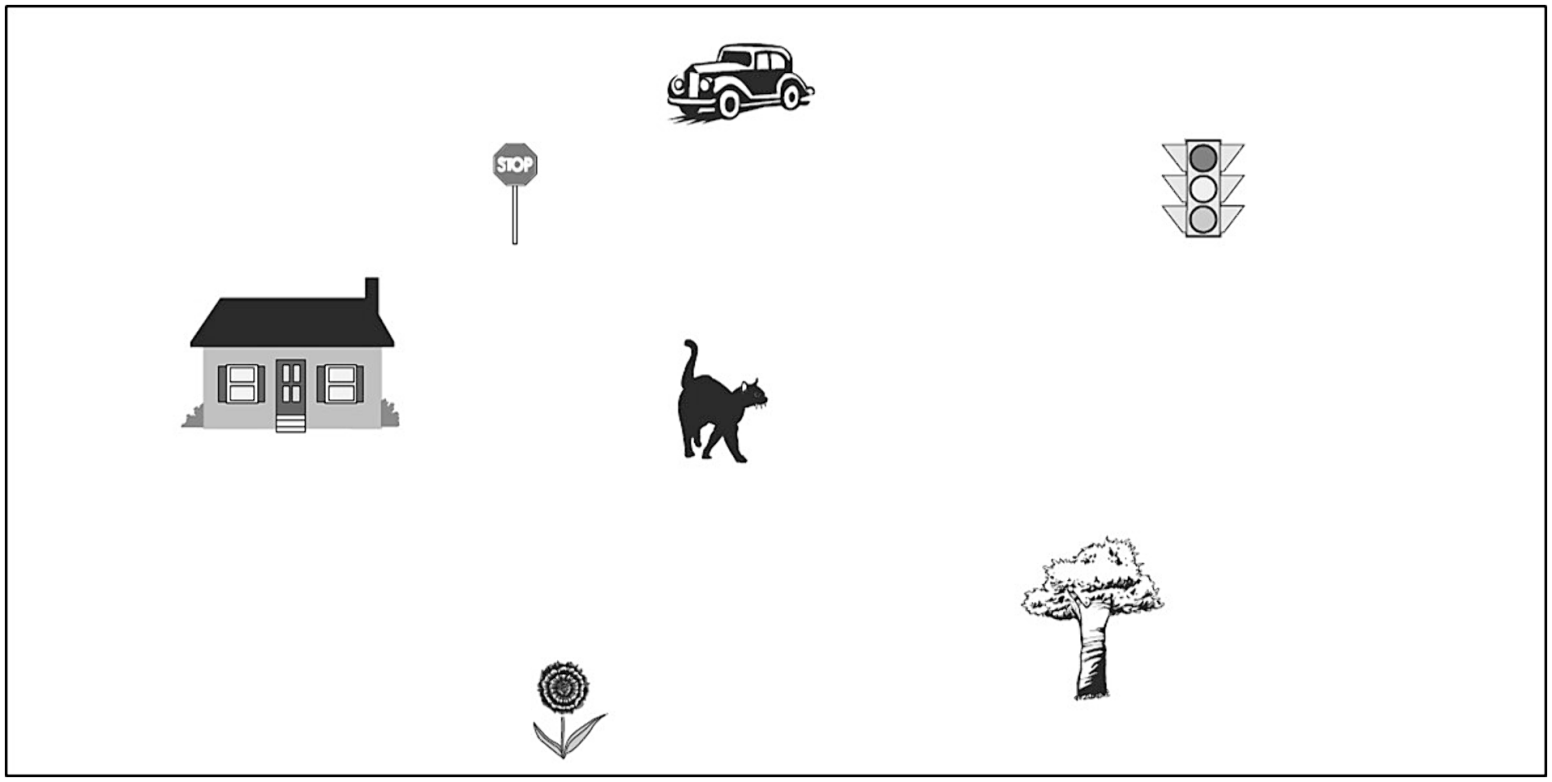
Display of the array of elements used for the sOPT task (adapted from Kozhevnikov and Hegarty, 2001; Hegarty and Waller, 2004).

The short Object Perspective-Taking (sOPT) task (adapted from Kozhevnikov and Hegarty, 2001; Hegarty and Waller, 2004) is a paper-and-pencil task devised for the assessment of visuospatial perspective taking skills. Children were given an A4 booklet, containing the six items and an explanation, together with a training item. The experimenter explained the task and how to respond; children were given 5 min to complete the task. Each page of the booklet contained an array of seven elements (Figure 1) on the top. For each item, instructions required children to imagine to be on one of the elements displayed in the array (station point), facing another element (imagined heading); they had to picture in their mind, where another element (target element) of the array would be. Participants had to mark it on a blank circle that in the A4 page was right below the array of elements. Correct responses were balanced, so that three were on the right side of the circumference and three on the left side. To compute the score, the experimenter calculated, for each item, the discrepancy (in degrees) between the correct and the given answer (i.e., angular disparity). The sum of these degrees constituted the score, the lower it was, the better the performance. Cronbach’s *α* = 0.79.

#### 2.2.2 Fine-motor skills

The supplementary motor task from the Beery-Buktenica Developmental Test of Visual-Motor Integration (VMI, Beery, 2004) was used to assess participants’ fine-motor coordination skills (i.e., movements of the hand and fingers). Children were presented with a booklet, containing the 27 items composing the task. Participants were asked to trace a given shape with a pencil, without going out the edges. A five-minute time was allowed for completing the task. Each correct item was awarded with one point. Raw scores were converted to age-appropriated standard scores (*M* = 100, DS = 15). Cronbach’s *α* = 0.82.

#### 2.2.3 Visual imagery

The Arrows task, taken from the NEPSY-II Battery (Korkman et al., 2007), was administered to assess visual imagery abilities (i.e., judgment of line orientation). For each item, participants were asked to look at an array of arrows displayed around a target and detect which of them pointed to its midpoint. The task comprised 21 items; according to the NEPSY-II Manual, an interruption criterion was applied in the case of five null answers (i.e., awarded with zero points) in a row. One point was given for each correct answer (maximum possible score: 38). Raw scores were considered. Cronbach’s *α* = 0.76.

#### 2.2.4 Mental rotation

The Letter Rotation task (adapted from Kaltner and Jansen, 2014) is a paper-and-pencil task devised for the assessment of mental rotation skills. Children were given an A4 booklet containing the 21 items composing the task, having 5 min to complete it. For each item, participants were presented with a 2D target figure (i.e., a letter) and were asked to identify, among four alternatives, the only picture that was a rotation (and had not been mirrored) of the target. One point was given for every correct answer. Accuracy was computed as the proportion of correct answers out of the total. Cronbach’s *α* = 0.91.

#### 2.2.5 Working memory

The computerized working memory task (adapted from Mammarella et al., 2018) was administered for the assessment of spatial-sequential working memory. Participants were presented with a 5×5 grid, displaying sequentially a series of black cells, varying between 2 and 8, according to each span level. Each item was available for 3 s, after which it was substituted with a blank grid, in which participants had to reproduce, by a click of the mouse, the pattern maintaining the order of presentation as well. The task embedded a self-terminating procedure, with a maximum number of 21 shown items (i.e., 3 items for each span level). Accuracy score was computed as the percentage of correct responses out of the total of the performed items, with higher scores reflecting a better performance (Giofrè and Mammarella, 2014). Cronbach’s *α* = 0.83.

### 2.3 Procedure

Each participant underwent two sessions, lasting approximately 60 min each, of individual testing. The sessions were arranged either in a quiet room at the clinical center where they were recruited, or in a laboratory provided by the University of Padua, Italy. Tasks were administered in a counterbalanced order, to mitigate the potential impact of fatigue on the results.

### 2.4 Data analysis

Data analysis were conducted using R [R Project for Statistical Computing (RRID:SCR_001905; RRID: SCR_000432; R Core Team, 2022)].

As a first step, aiming to highlight statistically significant differences between groups, descriptive statistics were obtained, and several univariate analyses of variance (ANoVA_s_) were computed. Bonferroni’s correction for multiple comparisons was used, when appropriate. In addition, for each comparison effect sizes (i.e., *η^2^_p_*) were computed.

Secondly, several linear models were built to detect statistically significant differences in the predictors that may account, for each of the considered groups, for the variance in the visuospatial perspective-taking performance.

A model selection strategy was adopted to identify the best-fitting model (e.g., Fox, 2008; Cardillo et al., 2020). This approach was adopted to enhance model interpretability, mitigate overfitting, and improve the generalization of findings by ensuring a more parsimonious and robust representation of the underlying relationships in the observed variables (see Burnham et al., 2011, for more details). A full model (M0) was computed, taking into account participants’ age and both fine-motor and visuo-spatial measures (i.e., visual imagery, mental rotation, and working memory) as well as the interactive effects between the Group and each of these variables. Aiming at highlighting such interactive effects between the Group and the variables, we applied a subtractive strategy, so that in the subsequent models (from M1 to M5) a predictor at a time was removed. For each of such obtained models, the Akaike Information Criterion (AIC, Akaike, 1998) and the relative likelihood (*l*) were computed. Following Burnham et al. (2011), the best-fitting model was identified as the one having the lowest AIC while *l* values higher than (or at) one were considered as indicative of a more plausible model as compared to models with *l* values smaller than 1. Moreover, *R^2^_adj_*, *F*, and *p* were computed for each model. Models are detailed in Table 2. Subsequently, the ANOVA function (“stats” package) was applied to the best fitting model (i.e., M0) aiming to compute the analysis of variance table for the linear model fits.

**Table 2:** Synthesis of the models considered in the model comparison procedure.

Models		*AIC*	*∆AIC*	*l*	*R^2^_adj_*	*F*	*p*
M0	sOPT ~ Group*(Age + FM + VI + MR + WM)	1135.987	0	1	0.355	3.722	<0.001
M1	sOPT~Age + Group*(FM + VI + MR + WM)	1140.793	−4.806	0.090	0.306	3.464	<0.001
M2	sOPT~Age + FM + Group*(VI + MR + WM)	1144.867	−8.879	0.012	0.258	3.245	<0.001
M3	sOPT~Age + FM + VI + Group*(MR + WM)	1144.290	−8.303	0.016	0.249	3.525	<0.001
M4	sOPT~Age + FM + VI + MR + Group*WM	1144.034	−6.248	0.018	0.236	3.877	<0.001
M5	sOPT~Group+Age + FM + VI + MR + WM	1142.235	−2.259	0.044	0.236	4.705	<0.001

AIC, Akaike Information Criterion; ∆AIC, difference between the full model’s AIC and the considered model’s AIC; *l*, relative likelihood. sOPT, visuospatial perspective-taking; FM, fine-motor; VI, visual imagery; MR, mental rotation; WM, spatial-sequential working memory.

## 3 Results

### 3.1 Group level differences

In Table 3 descriptive statistics and statistical comparisons (ANoVA_s_) between the three groups (i.e., DVSD, DCD, ND) are reported.

**Table 3:** Descriptive statistics by group and univariate analysis of the variance (ANoVAs): Developmental Visuospatial Disorder (DVSD), Developmental Coordination Disorder (DCD), and without any diagnosis (not-diagnosed, ND).

Measures	DVSD (*N* = 26) M (SD)	DCD (*N* = 26) M (SD)	ND (*N* = 33) M (SD)	*F*(2,82)	*p*	*η^2^_p_*	Group significance
Visuospatial perspective-taking	442.31 (225.27)	404.29 (210.43)	295.67 (195.14)	3.97	0.023	0.088	DVSD, DCD > ND
Fine-motor	83.50 (11.91)	86.65 (12.77)	99.58 (12.39)	13.14	<0.001	0.243	DVSD, DCD < ND
Visual imagery	25.69 (5.11)	27.31 (3.67)	31.76 (4.60)	14.56	<0.001	0.262	DVSD, DCD < ND
Mental rotation	0.71 (0.17)	0.85 (0.17)	0.93 (0.15)	13.27	<0.001	0.245	DVSD < DCD, ND
Working memory	18.67 (8.62)	25.65 (9.65)	37.52 (9.55)	31.12	<0.001	0.432	DVSD < DCD < ND

Only significant comparisons are reported. Visuospatial perspective-taking: sOPT (adapted from Kozhevnikov and Hegarty, 2001; Hegarty and Waller, 2004); Fine-motor: supplementary motor task from the VMI (Beery, 2004); Visual imagery: Arrows subtest from the NEPSY-II Battery (Korkman et al., 2007); Mental rotation: Letter Rotation task (adapted from Kaltner and Jansen, 2014); Working memory: spatial-sequential working memory task (adapted from Mammarella et al., 2018).

#### 3.1.1 Visuospatial perspective taking

A significant main effect of group was found for the sOPT task [*F*(2,82) = 3.97, *p* = 0.023; *η^2^_p_* = 0.088], with both the DVSD and DCD groups performing worse (i.e., higher degrees of error) than the ND group (respectively, *p* = 0.009 and *p* = 0.05). No significant difference emerged between the DVSD and the DCD groups (*p* = 0.515).

#### 3.1.2 Fine-motor skills

A significant main effect of group emerged for the supplementary motor task from the VMI [*F*(2,82) = 14.30, *p* < 0.001; *η^2^_p_* = 0.259], showing the ND group outperforming both the DVSD and the DCD groups (both *p_s_* < 0.001). No significant difference emerged between the DVSD and the DCD groups (*p* = 0.360).

#### 3.1.3 Visual imagery

In the Arrows task from the NEPSY-II Battery a significant main effect of group emerged [*F*(2,82) = 14.56, *p* < 0.001; *η^2^_p_* = 0.262]. Both the DVSD and DCD groups obtained lower scores than the ND group (both *p_s_* < 0.001). No significant difference emerged between the DVSD and the DCD groups (*p* = 0.200).

#### 3.1.4 Mental rotation

In the Letter Rotation task a main effect of group emerged [*F*(2,82) = 13.27, *p* = <0.001; *η^2^_p_* = 0.245], with lower scores for the DVSD group as compared to the DCD and ND groups (respectively, *p* = 0.002 and *p* < 0.001). No significant difference emerged between the DCD and the ND groups (*p* = 0.073).

#### 3.1.5 Working memory

A significant main effect of group emerged for the spatial-sequential working memory task [*F*(2,82) = 31.13, *p* < 0.001; *η^2^_p_* = 0.432]: the DVSD group obtained lower scores than both the DCD and the ND groups (respectively, *p* = 0.008 and *p* < 0.001). In addition, the DCD group’s scores were lower than those of the ND group (*p* < 0.001).

### 3.2 Linear regression model

As previously mentioned, to identify the best-fitting model, seven models were compared. Models are detailed in Table 2, with the full model (M0) showing the best fit.

**Figure 2:**
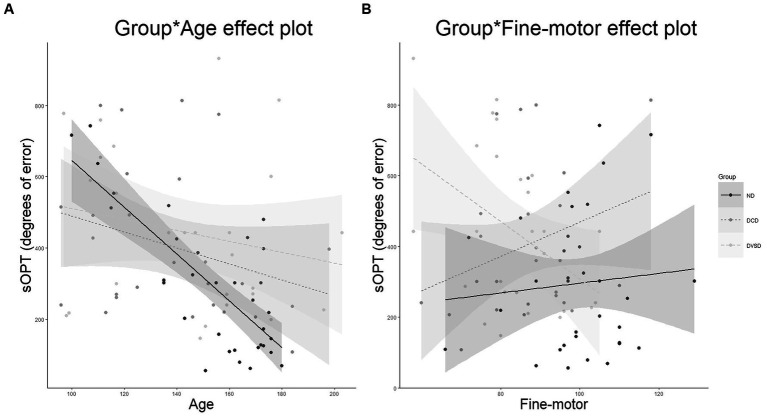
Significant effects of the best-fitting model for the performance at the sOPT (degrees of error). **(A)** is depicted the interaction between age and group, **(B)** the interaction between fine-motor abilities and group. DVSD, Developmental Visuo-Spatial Disorder; DCD, Developmental Coordination Disorder; ND, not diagnosed; sOPT, short Object Perspective-Taking task.

Specifically, in the linear regression model (Table 4), having the total degree of errors at the sOPT task as the dependent variable, the hypothesized predictors accounted for the 36% of the variance, calculated using the adjusted *R^2^* [*F*(17,67) = 3.72, *p* < 0.001]. Among the predictors, significant main effect of the group [*F*(2,67) = 5.74, *p =* 0.005] emerged. Moreover, a main effect of spatial-sequential working memory was observed [*F*(1,67) = 3.94, *p = 0*.05]: for all groups, higher scores in the working memory task predicted lower degrees of errors in the sOPT task. Moreover, two interaction effects were observed. First, a significant interaction between the group and age emerged: limited to the ND group [*F*(2,67) = 4.41, *p =* 0.016], older participants made fewer errors in the sOPT task (Figure 2A). The interaction between DVSD and DCD groups and the fine-motor task was also significant [*F*(2,67) = 3.54, *p = 0*.035], suggesting that for the DVSD group higher scores in the fine-motor task predicted a more accurate performance (i.e., lower degrees of error) in the sOPT task, while for the DCD group an opposite pattern emerged, with higher scores in the fine-motor task being associated to a worse performance in the sOPT task (Figure 2B).

**Table 4:** Regression analyses with spatial perspective-taking as dependent variable.

Predictors	*Df*	*F*	*p*	*η^2^_p_*
Group	2, 67	5.744	0.005	<0.001
Age	1, 67	19.925	<0.001	0.107
Fine-motor	1, 67	0.312	0.578	0.001
Visual imagery	1, 67	<0.001	0.995	0.006
Mental rotation	1, 67	3.361	0.071	0.022
Working memory	1, 67	3.940	0.05	0.031
Group*Age	2, 67	4.408	0.016	0.098
Group*Fine-motor	2, 67	3.537	0.035	0.096
Group*Visual imagery	2, 67	1.349	0.267	0.061
Group*Mental rotation	2, 67	1.103	0.338	0.020
Group*Working memory	2, 67	1.724	0.186	0.049

## 4 Discussion

Visuospatial perspective-taking has been proven to cover a relevant role in adaptive functioning (Frick and Baumeler, 2017), being related to domains such navigation and wayfinding, as well as to social relationships (Kozhevnikov et al., 2006; Hamilton et al., 2014; Tanaś and Myslinska Szarek, 2021). Different investigations have provided insights into how various underlying visuospatial (Kozhevnikov and Hegarty, 2001; Hegarty and Waller, 2004; Kessler and Rutherford, 2010; Meneghetti et al., 2012; Esenkaya et al., 2017; Cardillo et al., 2020) and motor skills (Cardillo et al., 2020; Hötting et al., 2021) contribute to supporting performance in VSPT.

Given this importance, it appears relevant to explore VSPT abilities in neurodevelopmental disorders, such as DVSD and DCD, characterized by core difficulties in visuospatial and motor domains, being the impairment in everyday functioning one of their main features (American Psychiatric Association, 2013).

Accordingly, the present study aimed to explore VSPT abilities in children with DVSD or DCD, as compared to a group of not-diagnosed peers (ND). Of interest was exploring the role that visuospatial skills, as well as fine motor skills, may cover in determining VSPT performance for each group. Firstly, considering the paucity of data on visuospatial perspective-taking in DVSD and DCD, possible differences between groups (i.e., DVSD, DCD, and ND) were analyzed. Then, the abilities underlying the performance in VSPT were investigated, taking into account both visuospatial processing and fine-motor predictors.

### 4.1 Group level differences

The analysis of the group comparisons ran on the performance in the VSPT test (i.e., the sOPT) revealed a significant difference in accuracy between the clinical groups as opposed to the ND peers. In fact, those latter participants’ performances were significantly more accurate than those of both the DVSD and DCD groups, resulting in lower degrees of error. This result is in line with previous descriptions of DVSD, which have highlighted the presence of core impairments in the visuospatial domain (Mammarella and Cornoldi, 2020; Fisher et al., 2022). As for the DCD group, our finding supports what previously concluded by Gauthier et al. (2018) who, albeit in the presence of methodological differences (i.e., in the task used), suggested the existence of an impairment in VSPT abilities in the DCD group as compared to typically developing peers. In this connection, it must be remarked that these authors pointed out that their DCD sample presented also with visuospatial impairments (Gauthier et al., 2018). On the other hand, since in the previous literature the comparison between DVSD and DCD has hardly ever been considered (Cardillo et al., 2024), the element of novelty of the present study is the comparison between the DVSD and DCD groups’ performance in their VSPT abilities. In this regard, it had been hypothesized a more severe impairment for the DVSD group, as compared to the DCD peers. Notably, our results are not consistent with such hypothesis, being the two groups’ scores in the VSPT task comparable. Although this task appears to apparently involve motor skills to a limited extent, in line with the corpus of research that argues that VSPT is itself based on a mechanism of motor embodiment (Amorim et al., 2006; Kessler and Thomson, 2010), we can hypothesize that the core motor impairment, hallmark of the DCD profile (American Psychiatric Association, 2013), could have played a role in determining the performance in the VSPT task for this group.

Considering the group comparisons ran on the other measures of visuospatial processing, as hypothesized (Mammarella and Cornoldi, 2020; Fisher et al., 2022; Mammarella et al., 2023) our results confirm the presence of generalized and severe impairments for the DVSD group (Mammarella et al., 2013, 2019; Basso Garcia et al., 2014), whose scores were significantly lower than those of the ND and DCD peers. On the other hand, consistently with our hypothesis (Bonifacci, 2004; Van Waelvelde et al., 2004; Tsai et al., 2008; Pisella et al., 2020), a more heterogeneous profile emerged for the DCD group. In fact, this latter group’s performances were alternatively in line with those of the DVSD or the ND group, or at the intermediate level between them, depending on the visuospatial domain examined. Considering visual imagery abilities, the DCD group’s scores were far below those of the ND sample and at the DVSD level, highlighting the presence of difficulties, even though the task used did not mean the involvement of motor skills. However, this result is in line with previous studies on visuo-spatial skills in DCD (Wilson and McKenzie, 1998; Prunty et al., 2016; Costini et al., 2017; Gomez and Huron, 2020). Conversely, the performance of the DCD group in the mental rotation task was comparable to that of the ND peers, and significantly better than that of the DVSD sample. This results is consistent with what previously observed on both children (Cardillo et al., 2024) and young adults (Barhoun et al., 2021) with a DCD diagnosis, suggesting the presence of spared mental rotation skills in this population. As regards visuo-spatial working memory abilities, the DCD group’s performance fell between those of the DVSD and ND groups. When comparing with the ND group, this outcome aligns with earlier observations that highlighted a working memory impairment in the DCD group (Alloway et al., 2009; Wang et al., 2017). The comparison between these two groups in this domain constitutes an element of novelty, since no previous study had directly compared DCD and DVSD peers in visuospatial working memory tasks. Nonetheless, there is still a need for future studies to better define the working memory profile in DVSD and DCD populations. The utility of adjunctive direct comparisons, run not only on spatial-sequential working memory but also on other components of the WM (e.g., spatial-simultaneous and verbal WM) (Mammarella et al., 2008) could contribute to shed more light on the peculiarities of the two profiles, thus allowing for a better characterization of each of them. Finally, as expected based on previous reports (Durand, 2005; American Psychiatric Association, 2013; Cardillo et al., 2024), the group comparison ran taking into account fine-motor (i.e., graph-motor) skills revealed comparable performances between the DCD and the DVSD groups. This result supports the stance of the presence of overlapping between neurodevelopmental disorders (Astle et al., 2022), yet being related to a task in which visuospatial processing skills might have played a role. To this extent, further investigations are required to assess the two groups’ abilities in purer measures of manual dexterity.

### 4.2 Visuospatial and fine-motor predictors to the VSPT performance

Stated the absence of statistically significant differences between the DVSD and DCD groups’ performances in the VSPT task, the second aim of the present study was to examine similarities and differences in the visuospatial and fine-motor predictors that might account for the performance in the visuospatial perspective taking task in our three groups. Considering the effect of age on the performance in the VSPT task (i.e., the sOPT), an interaction effect between this variable and group emerged. Consistently with Hodgkiss et al. (2021), but limited to the ND group, older participants’ performances in the VSPT task were more accurate. On the contrary, the effect of age was not significant either in the DVSD group nor for the DCD group. Stated the constitutional difficulties of these two clinical groups (Blank et al., 2019; Fisher et al., 2022), this finding might suggest that, unlike what has been observed for the ND group, growing older does not imply a significant increase in accuracy in the VSPT task for participants with either DVSD or DCD. On the other hand, but linked with this hypothesis, is the motivational factor. In fact, participants in the two clinical groups might have experienced a number of failures in tasks assessing visuospatial processing in their own story, thus leading them to avoid cognitive seffort in a task they perceived as exceeding their capabilities (Maier and Seligman, 1976). In line with previous reports on typical and atypical development (Meneghetti et al., 2012; Eilam and Alon, 2019; Cardillo et al., 2020), our results support the involvement of visuospatial working memory in the performance in the VSPT task (Meneghetti et al., 2012) for either the three groups. In other words, better abilities in WM were associated with better performances in the VSPT task, so that participants who committed a higher number of degrees of error had weaker WM abilities. Going along and beyond what found by Cardillo et al. (2020), our finding suggests that what was already known for the ND population might be extended to populations with neurodevelopmental disorders other than Autism spectrum disorder, such as DVSD and DCD. Indeed, previous data on either of the populations suggested the presence of visuospatial WM deficits (e.g., Basso Garcia et al., 2014; Wang et al., 2017). Although our results on group comparisons highlight differences between the DVSD and the DCD groups in this domain, what emerges from the linear model suggests that visuospatial WM may be associated with the poor performances observed by both groups in the VSPT task. In addition, fine-motor skills emerged as connected to the performance in the visuospatial perspective-taking task in both our clinical groups, but with an opposite pattern. As for the DVSD group, a positive association emerged between fine-motor abilities and the accuracy in the VSPT task (i.e., lower degrees of error); on the contrary, a worse performance in the VSPT task (i.e., higher degrees of error) was associated with higher scores in the fine-motor task for the DCD group. This result might suggest that children with DVSD might rely on fine-motor abilities while performing the VSPT task, while DCD participants might not take advantage of those skills. Nevertheless the absence of statistically significant differences between our DVSD and DCD samples in the fine-motor task, consistently with previous data (Cardillo et al., 2024), suggest that, even in the case of comparable abilities between groups, their performance in the sOPT may be associated with different mechanisms. Finally, in line with Cardillo et al. (2020), fine-motor abilities were not related to the performance in the VSPT task for the ND group. Surprisingly, and in contrast with previous studies conducted taking into account the typical population (Kozhevnikov and Hegarty, 2001; Hegarty and Waller, 2004), the effect of mental rotation abilities did not emerge as significant, neither in the ND group. It is worth noting that there is a trend toward significance for the main effect of mental rotation in predicting group performance on the VSPT task, even though the effect does not reach statistical significance. A possible explanation for this lack of effect might rely on the limited number of participants and the extreme variability of performance in our sample.

### 4.3 Limitations and future directions

To sum up, we have highlighted that the performance in a visuospatial perspective-taking task might be sustained by both shared and unshared predictors for different groups (i.e., DVSD, DCD and ND), thus suggesting the possible employment of different abilities by group. However, even though this is one of the first studies in which DVSD and DCD are directly compared, future research is needed to overcome its limitations.

First, despite presenting a cross-disorder comparison, our sample size is relatively limited, and the age range of participants is relatively high. These limitations entail two different considerations on the features of our groups. Firstly, the small sample size came because of the need to balance the requirements for conducting the research with the availability of practitioners and families of children diagnosed with DVSD or DCD. Moreover, as we pointed out while describing our participants, above and beyond the presence of a clinical diagnosis, we recurred to strict inclusion and exclusion criteria, comprising, for instance, the exclusion of those participants whose profiles evoked the co-occurrence of DVSD and DCD. Taking into account the complexity of such defined framework, we were unable to run an *a-priori* power analysis. At the same time, we have not deemed to run a retrospective power analysis, in light of it being generally considered uninformative (Hoenig and Heisey, 2001). Secondly, it must be noted that, according to the gender distribution in neurodevelopmental disorders (American Psychiatric Association, 2013), our sample comprised a higher number of males than females. The limited number of females across our groups prevented us considering this variable as a predictor in the regression model, though future studies could shed light on the possible effect of gender on performances in the visuospatial domain in these neurodevelopmental conditions (Lanzenberger et al., 2020).

Second, it must be considered that the fine-motor task we have used is aimed at the assessment of graph-motor coordination. In this sense, although the VSPT task we have used requires children to recur to their graph-motor abilities to mark their answer (Cardillo et al., 2020), it might be useful to take into account also other manual dexterity measures. In addition, given the complexity of the request that the VSPT task posits, it may somehow be considered that cognitive skills other than those we have considered may cover a role in determining the performance at the task (Fizke et al., 2014; Qureshi and Monk, 2018; Samuel et al., 2023).

Finally, as concerns the possibility of further examine perspective-taking abilities in developmental populations, it may be useful to take into account not only the performance in terms of accuracy, but also the strategies participants used to solve the task (Meneghetti et al., 2012). This aspect may become particularly relevant in the perspective of setting up interventions to sustain VSPT abilities in children, especially when populations with neurodevelopmental disorders are involved. In fact, being VSPT linked to everyday adaptive functioning (Allen et al., 1996; Kozhevnikov et al., 2006; Hamilton et al., 2014; Tanaś and Myslinska Szarek, 2021), getting to know specific impairments and strategies by group may cover a predominant role in designing interventions specifically tailored on the needs of each population. In fact, extending what stated by Semrud-Clikeman et al. (2010), interventions appear to be more effective when built taking into account not only the overall performance, but also the reasons underlying that performance.

## 5 Conclusion

In conclusion, the present study adds to the extant literature a contribution on the knowledge on VSPT abilities in DVSD and DCD, two conditions that have hardly ever been compared, although presenting with some overlapping traits. Of interest was the direct comparison between these groups and a ND group on a measure of VSPT. Our results suggest that the two clinical groups’ performances were not significantly different one from the other, albeit being significantly less accurate than those of the ND peers. Nonetheless, among the abilities that were related to the performance in the VSPT domain, both similarities and differences by group were observed. In fact, visuospatial working memory emerged as associated with the VSPT performance in all the three groups, while different effects of age and fine-motor skills emerged as specific for some given groups. Age seemed to be related to a better performance only for the ND group, while opposite patterns emerged for the involvement of fine-motor skills in DVSD and DCD. However, further research is needed to better define the features and to refine the most sensitive diagnostic tools for both DVSD (Fisher et al., 2022) and DCD (Blank et al., 2019; Zoia et al., 2022).

## Data availability statement

The datasets analyzed during the current study are not publicly available due to ethics and privacy issues involving participant’s data but will be made available from the corresponding author on request.

## Ethics statement

Ethical approval was obtained from the Research Ethics Committee (area 17) at the University of Padua, Italy. The study was conducted in accordance with the local legislation and institutional requirements. Written informed consent for participation in this study was provided by the participants’ legal guardians/next of kin.

## Author contributions

CO: Formal analysis, Investigation, Methodology, Writing – original draft, Writing – review & editing. RC: Conceptualization, Formal analysis, Investigation, Methodology, Writing – review & editing. IL: Resources, Writing – review & editing. LZ: Resources, Writing – review & editing. ICM: Conceptualization, Funding acquisition, Supervision, Writing – review & editing.
